# Glutathione S-transferase gene polymorphisms and risk of nasal or colorectal polyposis

**DOI:** 10.1042/BSR20181226

**Published:** 2019-01-25

**Authors:** Yonglan Zhang, Haichao Zhang, Peng Lin, Guimin Zhang

**Affiliations:** 1 Department of Otolaryngology-Head and Neck Surgery, Tianjin First Center Hospital, Tianjin 300192, People’s Republic of China; 2 Department of Thyroid and Breast Surgery, Tianjin 4th Center Hospital, Tianjin 300140, People’s Republic of China

**Keywords:** GSTM1, GSTT1, GSTP1, nasal polyposis, polymorphism

## Abstract

We observed inconsistent conclusions regarding the genetic role of glutathione S-transferase gene polymorphisms, including glutathione S-transferase M1 (*GSTM1*), glutathione S-transferase T1 (*GSTT1*) present/null, and glutathione S-transferase pi *(GSTP1*) Ile105Val polymorphisms, in the susceptibility to nasal or colorectal polyposis (NP or CP). Thus, we aimed to perform a meta-analysis to comprehensively evaluate this association by applying Stata/SE software. After the heterogeneity assumption, Mantel–Haenszel statistics were used to obtain the odds ratio (OR), 95% confidence interval (95% CI) and *P*-value of the association test (*P_A_*). We obtained a total of 235 articles by searching online databases. After screening, ten eligible case–control studies were finally enrolled in our meta-analysis. For the meta-analysis of the *GSTT1* gene under present versus null, we observed a decreased risk of NP [OR = 0.65; *P_A_*=0.018], but not CP. In addition, we did not detect any evident association between the *GSTM1* present/null polymorphism and NP or CP risk. For the meta-analysis of the *GSTP1* Ile105Val polymorphism, compared with controls, an increased risk of NP cases was detected under the models of Val versus Ile (OR = 1.36; *P_A_*=0.027), Ile/Val versus Ile/Ile (OR = 1.70; *P_A_*=0.011) and Ile/Val+Val/Val versus Ile/Ile (OR = 1.65; *P_A_*=0.010). In conclusion, the null genotype of the *GSTT1* polymorphism may be linked to an increased susceptibility to NP, whereas the Ile/Val genotype of the *GSTP1* Ile105Val polymorphism may be associated with a decreased risk of NP.

## Introduction

Polyposis refers to a chronic disorder characterized by the presence of polyps, which are neoplasms that grow on the mucosal surface of human organs or tissues, including the nasal cavity, vocal cord, stomach, or colorectal area [1–3]. The type of polyposis (e.g., nasal polyposis or colorectal polyposis [NP or CP], etc.) is named according to its location [1–3]. NP, a chronic and complicated inflammatory disorder with the formation of mostly benign polyps within bilateral nasal cavities, encompasses a series of pathological features, such as epithelial cell proliferation, eosinophil infiltration, and glandular changes [4,5]. Colorectal polyps are mostly regarded as a type of benign tumor, but some show malignant tendencies, and hence the removal of polyps is an effective approach to prevent the occurrence of cancer [6].

Although the exact etiology of polyposis remains to be elucidated, chronic stimulation and genetic factors may partly account for the presence of polyps [7,8]. Genetic variants reportedly involved in the complicated etiology or pathogenesis of NP or CP are increasingly reported [7,9,10].

The proteins glutathione S-transferase M1 (GSTM1), glutathione S-transferase T1 (GSTT1), and glutathione S-transferase pi (GSTP1), which are encoded by the *GSTM1*, *GSTT1*, and *GSTP1* genes, respectively, are the three most common classes (mu, theta, and pi) of glutathione S-transferases in the human body [11,12]. The present/null polymorphism is the common variant of the *GSTM1* and *GSTT1* genes, while Ile105Val (rs1695) and Ala114Val (rs1138272) are the two common polymorphisms of the *GSTP1* gene [11,12]. Conflicting conclusions on the associations between polymorphisms of *GSTM1, GSTT1*, and *GSTP1* and the risk of NP or CP have been reported [12–21]. No specific meta-analysis of this topic has been performed. Thus, we are interested in quantitatively examining such an association by pooling published related evidence together.

## Materials and methods

### Database search strategy

We retrieved articles from four electronic databases, PubMed, Web of Science (WOS), Embase, and China National Knowledge Infrastructure (CNKI), published up to January 2018. For example, the PubMed database was searched using the following medical subject heading (MeSH) terms: (((((((((((Polyps[MeSH Terms]) or Polyp) or Adenomatous Polyps) or Intestinal Polyps) or Colonic Polyps) or Nasal Polyps) or Intestinal Polyp) or Colonic Polyp) or Nasal Polyp) or Adenomatous Polyp)) and ((((((((((((((((Glutathione S-Transferase pi[MeSH Terms]) or Glutathione S Transferase pi) or GST Class-phi) or Class-phi, GST) or GST Class phi) or Glutathione Transferase P1-1) or Glutathione Transferase P1 1) or Transferase P1-1, Glutathione) or GSTP1 Glutathione D-Transferase) or D-Transferase, GSTP1 Glutathione) or GSTP1 Glutathione D Transferase) or Glutathione D-Transferase, GSTP1) or GSTP1)) or ((((((((Glutathione S-transferase M1[MeSH Terms]) or Gstm1 protein, mouse) or glutathione S-transferase, mu 1 protein, mouse) or Gstm1 protein, rat) or glutathione S-transferase, mu 1 protein, rat) or GSTM1 protein, human) or glutathione S-transferase M1, human) or GSTM1)) or ((((((((((glutathione S-transferase T1[MeSH Terms]) or glutathione S transferase theta 1) or glutathione S transferase GSTT1) or Gstt1 protein, mouse) or glutathione S-transferase, theta 1 protein, mouse) or Gstt1 protein, rat) or glutathione S-transferase theta 1, rat) or GSTT1 protein, human) or glutathione S-transferase theta 1 protein, human) or GSTT1)).

### Study screening strategy

We designed our screening strategy according to the ‘Preferred Reporting Items for Systematic Reviews and Meta-Analyses (PRISMA)’ [22]. The main exclusion criteria were as follows: (1) review or meta-analysis; (2) meeting abstract; (3) other genes/diseases; (4) non-polymorphism data; (5) cell data; (6) data without genotype; and (7) duplicated studies. For eligible case–control studies, the complete genotype frequency data of *GSTM1*, *GSTT1*, and *GSTP1* gene variants in both polyposis cases and negative controls were available.

### Data extraction strategy

We thoroughly extracted the following basic information from each retrieved case–control study via a predesigned table: first author name, publication year, country, ethnicity, gene, present/null frequencies of the *GSTM1* and *GSTT1* genes, the Ile/IIe, Ile/Val, and Val/Val genotype frequencies of the *GSTP1* Ile105Val polymorphism, disease type, genotyping method, source of control, and sample size. We also evaluated the study quality and asked for missing data by emailing the original authors.

### Statistical analysis

We used Stata/SE software (StataCorp, U.S.A.) for our statistical analysis. A fixed-effect model was used for Mantel–Haenszel statistics when the *P*-value of heterogeneity from Cochran’s *Q* statistic >0.1 or I^2^ value <50.0%. We obtained the value of the summary odds ratio (OR), 95% confidence intervals (95% CIs), and *P*-value of the association test (*P_A_*). The* P_A_* of less than 0.05 was deemed statistically significant. The present versus null genetic model was used for the *GSTM1* and *GSTT1* genes, and the allele (Val versus Ile), homozygote (Val/Val versus Ile/Ile), heterozygote (Ile/Val versus Ile/Ile), dominant (Ile/Val+Val/Val versus Ile/Ile), and recessive (Val/Val versus Ile/Ile+Ile/Val) models were used for the *GSTP1* gene.

We performed subgroup meta-analyses according to ethnicity (Caucasian/Asian), disease type (NP/CP), source of control (population/hospital-based), and country (The Netherlands/Germany). We also performed the Begg’s/Egger’s tests to assess the publication bias among the selected studies and sensitivity analyses to measure the statistical stability of the results.

## Results

### Case–control study inclusion

**Figure 1:**
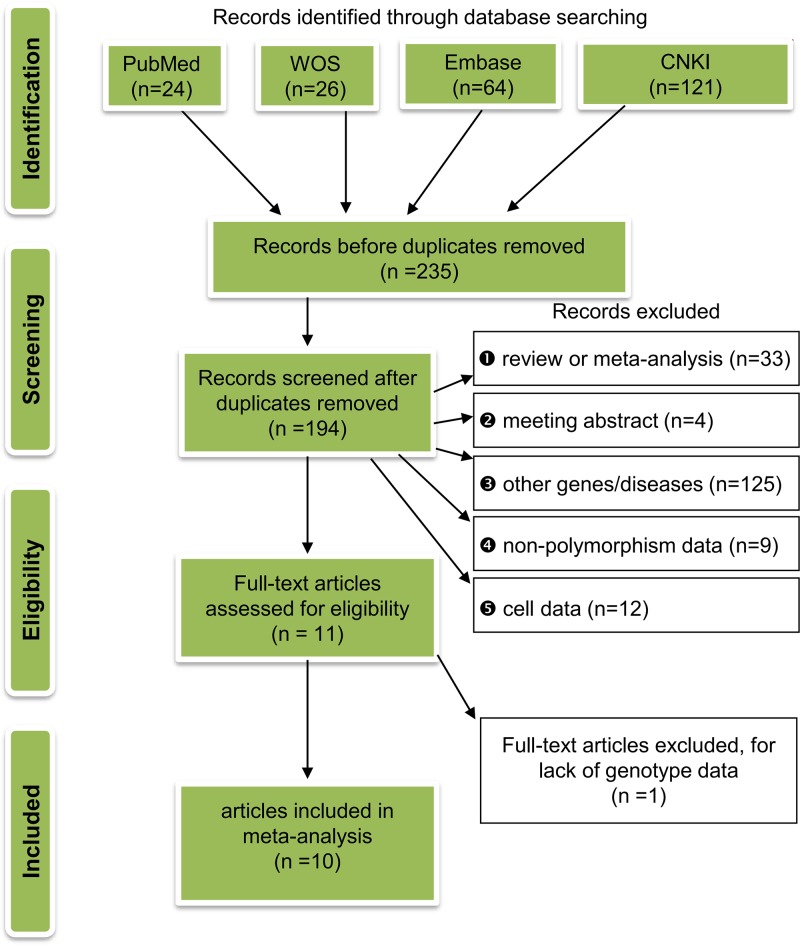
Flow chart for inclusion of eligible case–control studies

As per the flow diagram in Figure 1, we obtained eligible case–control studies for our meta-analysis. First, we retrieved a total of 235 records (24 records from PubMed, 26 records from WOS, 64 records from Embase, and 121 records from CNKI) by searching online databases. Second, we excluded 41 records because of duplication and another 194 records due to the exclusion criteria (details shown in Figure 1). Third, after assessing the 11 remaining full-text articles for eligibility, we further ruled out one article without genotype frequency data. Ultimately, ten eligible articles [12–21] were collected. Table 1 shows the basic information of the case–control studies. The present/null polymorphisms of the *GSTM1* and *GSTT1* genes and the Ile105Val polymorphism of the *GSTP1* gene were tested.

**Table 1:** Basic information of case–control studies

First author, year [REF]	Country	Ethnicity	Gene	Case			Control			Method
				Total	Present/null	Disease	Total	Present/null	Source	
Arbag, 2006 [18]	Turkey	Asian	*GSTM1*	98	55/43	NP	102	55/47	PB	PCR
			*GSTT1*	98	65/33	NP	102	78/24	PB	PCR
Berkhout, 2008 [19]	The Netherlands	Caucasian	*GSTM1*	85	38/47	CP	215	100/115	PB	PCR
			*GSTT1*	85	60/25	CP	214	165/49	PB	PCR
			*GSTP1*	85	31/39/15^#^	CP	209	90/86/33^#^	PB	PCR
Fruth, 2011 [15]	Germany	Caucasian	*GSTM1*	69	34/35	NP	49	23/26	HB^1^	PCR
			*GSTM1*	69	34/35	NP	52	23/29	HB^2^	PCR
			*GSTT1*	69	52/17	NP	49	36/13	HB^1^	PCR
			*GSTT1*	69	52/17	NP	52	42/10	HB^2^	PCR
			*GSTP1*	69	27/28/14^#^	NP	49	28/16/5^#^	HB^1^	PCR
			*GSTP1*	69	27/28/14^#^	NP	52	27/18/7^#^	HB^2^	PCR
Gawronska, 1999 [13]	Poland	Caucasian	*GSTM1*	27	10/17	CP	145	73/72	PB^3^	PCR
			*GSTM1*	27	10/17	CP	17	8/9	HB^4^	PCR
Hamachi, 2013 [21]	Japan	Asian	*GSTM1*	455	200/255	CP	1052	506/546	HB	Multiplex PCR
			*GSTT1*	455	258/197	CP	1052	552/500	HB	Multiplex PCR
Lamberti, 2002 [20]	Germany	Caucasian	*GSTM1*	402	185/217	CP	171	77/94	PB	Multiplex PCR
			*GSTM1*	402	185/217	CP	200	99/101	NR	Multiplex PCR
			*GSTT1*	406	336/70	CP	172	144/28	PB	Multiplex PCR
			*GSTT1*	406	336/70	CP	148	125/23	NR	Multiplex PCR
Lin, 1998 [17]	U.S.A.	Mixed	*GSTM1*	459	255/204	CP	507	258/249	HB	PCR
Ozcan, 2010 [12]	Turkey	Asian	*GSTM1*	75	45/30	NP	167	92/75	PB	PCR
			*GSTT1*	75	54/21	NP	167	142/25	PB	PCR
			*GSTP1*	75	28/37/10^#^	NP	167	77/59/31^#^	PB	PCR
Tiemersma, 2004 [14]	The Netherlands	Caucasian	*GSTM1*	431	203/228	CP	432	206/226	HB	Multiplex PCR
			*GSTT1*	431	370/61	CP	432	363/69	HB	Multiplex PCR
Tijhuis, 2005 [16]	The Netherlands	Caucasian	*GSTM1*	746	363/383	CP	698	320/378	HB	Multiplex PCR
			*GSTT1*	746	612/134	CP	698	571/127	HB	Multiplex PCR

# , genotype frequency of GSTP1 gene (IIe/IIe, Ile/Val, Val/Val); ^1^, chronic rhinosinusitis without nasal polyps; ^2^, healthy tissue controls of inferior turbinate; ^3^, healthy individuals; ^4^, hereditary non-polyposis colorectal cancer.Abbreviations: HB, hospital-based control; NR, not reported; PB, population-based control; REF, reference.

### *GSTM1* and *GSTT1* polymorphism

We first performed a meta-analysis to study the genetic relationships between the present/null polymorphisms of the *GSTM1* and *GSTT1* genes and the risk of polyposis. As shown in Table 2, there was no high degree of heterogeneity in the present versus null model of *GSTM1* (I^2^ value = 0.0%, *P*-value of Cochran’s *Q* statistic test [*P_H_*] =0.686, and *GSTT1* [I^2^ value = 31.4%, *P_H_*=0.157]); consequently, a fixed-effect model was selected for the Mantel–Haenszel statistics. As shown in Table 2, 3345 cases and 3807 controls were included for the quantitative synthesis of the *GSTM1* present/null polymorphism, while 2840 cases and 3086 controls were available for the *GSTT1* polymorphism. In the pooled estimates of the overall meta-analysis, no difference between the case and control groups for the risk of polyposis was observed for *GSTM1* [Table 2, *P_A_*=0.838] and *GSTT1* (*P_A_*=0.914).

**Table 2:** Overall meta-analysis of the *GSTP1, GSTM1, GSTT1* polymorphism and the risk of polyposis

Gene	Genetic models	I^2^	*P_H_*	Fixed/random	OR	95% CI	*P*_A_	*P*_B_	*P*_E_	Case/control
*GSTM1*	Present vs null	0.0%	0.686	Fixed	1.01	0.92–1.11	0.838	0.583	0.612	3345/3807
*GSTT1*	Present vs null	31.4%	0.157	Fixed	0.99	0.87–1.13	0.914	0.032	0.016	2840/3086
*GSTP1*	Val vs Ile	3.9%	0.373	Fixed	1.30	1.04–1.62	***0.019***	0.308	0.094	298/477
	Val/Val vs Ile/Ile	4.9%	0.368	Fixed	1.44	0.93–2.24	0.101	0.308	0.217	298/477
	Ile/Val vs Ile/Ile	0.0%	0.897	Fixed	1.55	1.12–2.16	***0.009***	0.734	0.444	298/477
	Ile/Val+Val/Val vs Ile/Ile	0.0%	0.784	Fixed	1.52	1.12–2.07	***0.007***	0.089	0.060	298/477
	Val/Val vs Ile/Ile+Ile/Val	0.0%	0.775	Fixed	1.22	0.95–1.58	0.123	0.308	0.069	298/477

Data in bold are *P_A_*<0.05. Abbreviaition: *P_E_, P-*value of Egger’s test.

**Table 3:** Subgroup meta-analysis of the *GSTM1, GSTT1* polymorphism and the risk of polyposis

Gene	Genetic models	Subgroup	OR	95% CI	*P*_A_	Case/control
*GSTM1*	Present vs null	NP	1.16	0.85–1.58	0.356	311/370
		CP	1.00	0.90–1.10	0.931	3034/3437
		PB	1.00	0.81–1.26	0.936	687/800
		HB	1.03	0.92–1.15	0.640	2256/2807
		The Netherlands	1.05	0.90–1.23	0.537	1262/1345
		Germany	0.98	0.79–1.23	0.883	942/472
		Asian	0.91	0.75–1.11	0.351	628/1321
		Caucasian	0.98	1.01–1.14	0.877	2258/1979
*GSTT1*	Present vs null	NP	0.65	0.45–0.93	***0.018***	311/370
		CP	1.06	0.92–1.21	0.431	3034/3437
		PB	0.70	0.53–0.93	***0.014***	687/800
		HB	1.11	0.95–1.29	0.189	2256/2807
		The Netherlands	1.01	0.82–1.24	0.934	1262/1345
		Germany	0.91	0.67–1.23	0.534	942/472
		Asian	1.02	0.83–1.214	0.870	628/1321
		Caucasian	0.98	0.82–1.16	0.779	2258/1979

Data in bold are *P_A_*<0.05.Abbreviations: PB, population-based control; HB, hospital-based control.

**Figure 2:**
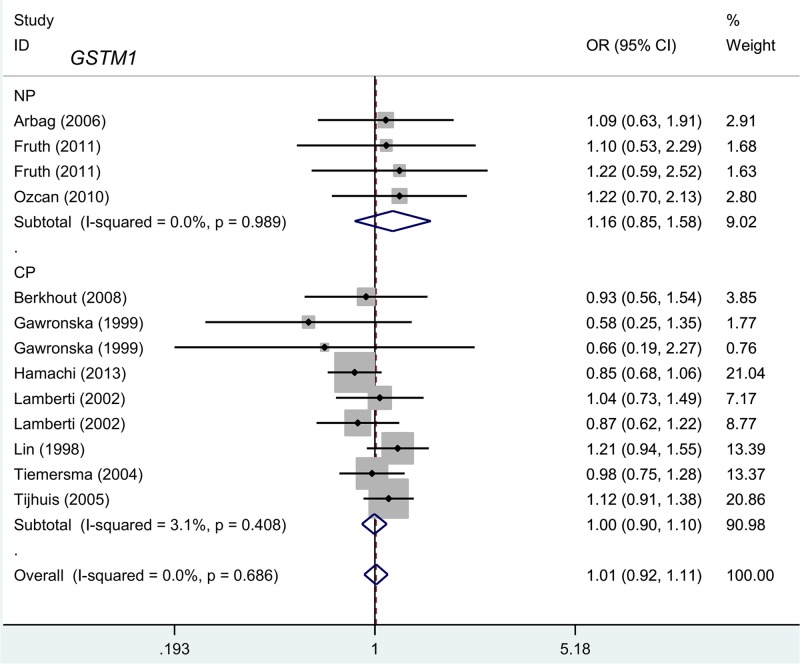
Subgroup analysis by disease type for the *GSTM1* polymorphism

**Figure 3:**
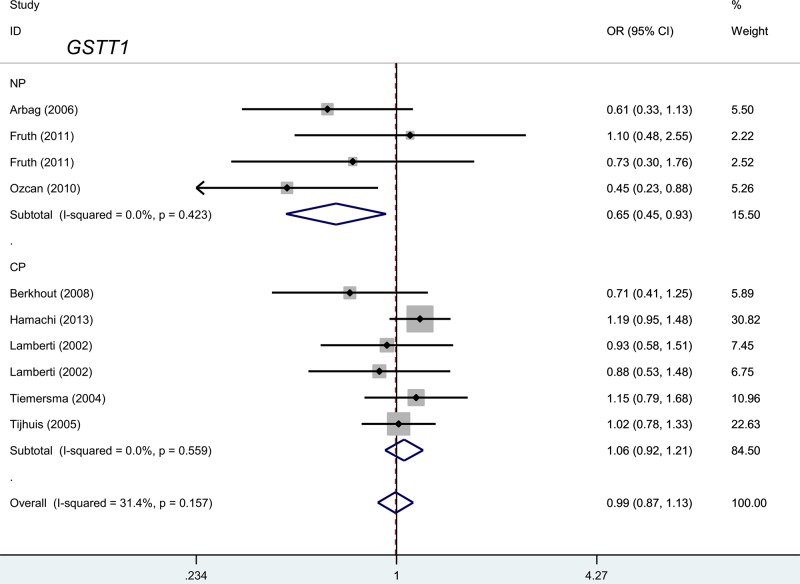
Subgroup analysis by disease type for the *GSTT1* polymorphism

Furthermore, we performed subgroup meta-analyses by disease type (NP/CP), source of control (population/hospital-based), country (The Netherlands/Germany), and ethnicity (Caucasian/Asian) for both the *GSTM1* and *GSTT1* genes. As shown in Table 3, a decreased risk of NP was detected for the *GSTT1* gene in the subgroups ‘NP", OR = 0.65, 95% CI = 0.45–0.93, *P_A_*=0.018) and ‘population-based’ (OR = 0.70, 95% CI = 0.53–0.93, *P_A_*=0.014) under the present versus null model. No differences between the cases and controls were observed for the other subgroups (all *P_A_*>0.05). We also constructed forest plots of the subgroup analysis by disease type for the *GSTM1* (Figure 2) and *GSTT1* (Figure 3) genes. These plots indicated that the *GSTT1* null genotype may be associated with susceptibility toward NP.

### *GSTP1* polymorphism

Next, we performed the overall analysis involving 298 cases and 477 controls for the association between the *GSTP1* Ile105Val polymorphism and polyposis susceptibility (Table 2). Mantel–Haenszel statistics with a fixed model was used (Table 2, all *I^2^*<50.0%, *P_H_*>0.1). We observed an increased polyposis risk in the models of Val versus Ile (OR = 1.30; 95% CI = 1.04–1.62; *P_A_*=0.019), Ile/Val versus Ile/Ile (OR = 1.55, 95% CI = 1.12–2.16, *P_A_*=0.009) and Ile/Val+Val/Val versus Ile/Ile (OR = 1.52, 95% CI = 1.12–2.07, *P_A_*=0.007) but not in the other genetic models (all *P_A_*>0.05). We obtained a similar conclusion for the subgroup ‘NP’ under the allele (Table 4, OR = 1.36, *P_A_*=0.027), heterozygote (OR = 1.70, *P_A_*=0.011) and dominant (OR = 1.65, *P_A_*=0.010) models. Moreover, we detected increased risk in the subgroups ‘*P-*value of Hardy–Weinberg Equilibrium test (*P*_HWE_) >0.05’ and ‘Caucasian’ only in the allele and homozygote models (Table 4, OR > 1, *P_A_*<0.05). Consequently, the Ile/Val genotype of *GSTP1* Ile105Val polymorphism is more likely to be associated with an increased risk of NP.

**Table 4:** Subgroup meta-analysis of the *GSTP1* Ile105Val polymorphism and the risk of polyposis

Genetic models	Subgroup	OR	95% CI	*P*_A_	Case/control
Val vs Ile	NP	1.36	1.04–1.80	***0.027***	213/268
	*P*_HWE_>0.05	1.41	1.08–1.84	***0.011***	223/310
	Caucasian	1.41	1.08–1.84	***0.011***	223/310
Val/Val vs Ile/Ile	NP	1.52	0.88–2.62	0.136	213/268
	*P*_HWE_>0.05	1.76	1.04–2.98	***0.034***	223/310
	Caucasian	1.76	1.04–2.98	***0.034***	223/310
Ile/Val vs Ile/Ile	NP	1.70	1.13–2.57	***0.011***	213/268
	*P*_HWE_>0.05	1.48	1.00–2.21	0.052	223/310
	Caucasian	1.48	1.00–2.21	0.052	223/310
Ile/Val+Val/Val vs Ile/Ile	NP	1.65	1.13–2.41	***0.010***	213/268
	*P*_HWE_>0.05	1.13	0.94–1.35	0.191	223/310
	Caucasian	1.13	0.94–1.35	0.191	223/310
Val/Val vs Ile/Ile+Ile/Val	NP	1.27	0.92–1.76	0.141	213/268
	*P*_HWE_>0.05	1.29	0.95–1.76	0.109	223/310
	Caucasian	1.29	0.95–1.76	0.109	223/310

Data in bold are *P_A_*<0.05.Abbreviation: *P*_HWE_, *P-*value of Hardy–Weinberg Equilibrium test.

### Publication bias and sensitivity analysis

**Figure 4:**
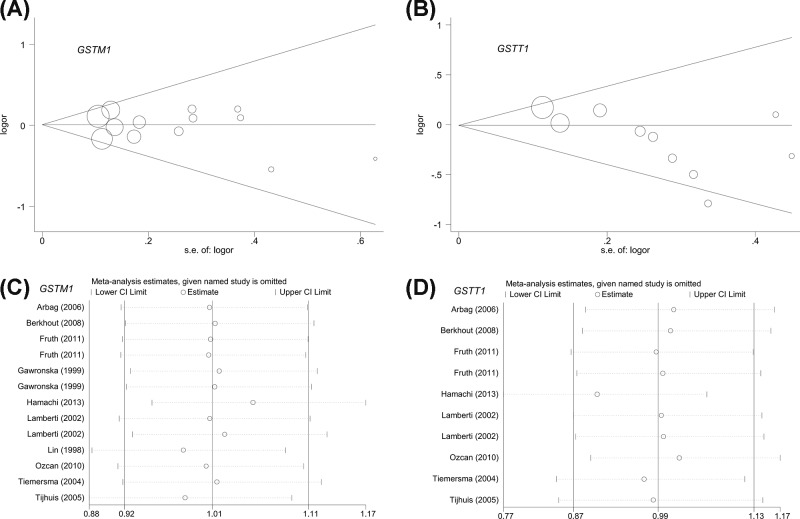
Begg’s test and sensitivity analysis for the *GSTM1* and *GSTT1* polymorphisms (**A** and **B**) Funnel plot of Begg’s test. (**C** and **D**) Sensitivity analysis.

We performed the Begg’s/Egger’s tests to evaluate publication bias. As shown in Table 2, we did not find evidence of a high degree of publication bias under all genetic models (*P-*value of Begg’s test [*P_B_*] >0.05; *P-*value of Egger’s test (*P_E_*) >0.05], with the exception of the present versus null model of the *GSTT1* gene (*P_B_*=0.032*, P_E_*=0.016). We also constructed funnel plots of Begg’s test under the present versus null model of the *GSTM1* (Figure 4A) and *GSTT1* (Figure 4A) genes.

Furthermore, we performed a sensitivity analysis to observe the statistical stability of the above conclusions (Figure 4C for *GSTM1*, Figure 4D for *GSTT1*, others not shown).

## Discussion

NP is thought to be closely associated with a group of clinical disorders characterized by chronic rhinosinusitis and shows differences in complexity and etiology [1,23,24]. A family-based genome-wide association study reported that the holocarboxylase synthetase (*HLCS*), major histocompatibility complex, class II, DR α (*HLA-DRA*), BICD cargo adaptor 2 (*BICD2*), V-set immunoregulatory receptor (*VSIR*), and solute carrier family 5 member 1 (*SLC5A1*) genes may be linked to the pathogenesis of chronic rhinosinusitis with nasal polyps [9]. Another study reported that NP risk is significantly associated with the present/null polymorphism of the *GSTT1* gene but not the present/null polymorphism of the *GSTM1* gene and the Ile105Val polymorphism of the *GSTP1* gene in the Mersin region of Turkey [12]. Conversely, in the Konya region of Turkey, a lack of genetic impact of the *GSTM1* and *GSTT1* polymorphisms on the predisposition for NP was reported [18]. There is statistical correlation between the polymorphisms of the *GSTT1*, *GSTM1*, and *GSTP1* genes and the genetic tendency of chronic rhinosinusitis with or without nasal polyps in Germany [15]. Accordingly, it would be of interest to evaluate the overall effect of the underlying roles of the *GSTM1* present/null*, GSTT1* present/null, and *GSTP1* Ile105Val polymorphisms in the susceptibility towards NP or CP . Our findings suggest that the null genotype of the *GSTT1* polymorphism and the *GSTP1* Ile105Val polymorphism are more likely to be associated with the risk of NP. However, no association between the *GSTM1* present/null polymorphism and the risk of NP or CP was observed.

Human glutathione S-transferases are a family of multifunctional enzymes with antioxidant activity that are involved in oxidative stress, cell differentiation, inflammatory responses, drug detoxification, and chemotherapy resistance [25]. Some meta-analyses have reported different conclusions regarding the association between these variants and clinical disease risk. For instance, the *GSTP1* Ile105Val polymorphism and *GSTM1* null genotype but not the *GSTT1* null genotype seem to increase the risk of Alzheimer’s disease [11]. Similarly, the *GSTM1* null genotype but not the *GSTT1* null genotype may be linked to the risk of juvenile open-angle glaucoma (JOAG) [26]. However, the null genotypes of the *GSTM1* and *GSTT1* genes and the combined *GSTM1*/*GSTT1* gene may be risk factors for endometriosis [27]. Our meta-analysis data supports a genetic role of the *GSTT1* null genotype and the Ile/Val genotype of the *GSTP1* Ile105Val polymorphism in the risk of NP. Individuals with the *GSTT1* null genotype may show partial gene deletion, followed by enzymatic activity deficiency and decreased detoxification capacity. The Ile105Val polymorphism of the *GSTP1* gene leads to an amino acid change from Ile to Val at residue 105 of the GSTP1 protein, which also may decrease the catalytic activity of the enzyme. Such a change may reduce the efficient detoxification of stress-induced intermediates, which has been implicated in the presence of an inflammatory response and nasal polyps in response to increased levels of electrophilic compounds or reactive oxygen species.

There are some limitations of the present study that should be fully discussed. We endeavoured to search relevant publications by using search terms for different polyps, such as adenomatous polyps, intestinal polyps, colonic polyps, nasal polyps, and intestinal polyps, without language or region restrictions. However, only the data on nasal and colorectal polyps permitted synthesis of the data. Detailed data on genotype frequencies in both cases and controls are required to perform the overall meta-analysis and subsequent subgroup analyses. Due to restrictions of data availability, only the Ile105Val polymorphism was analyzed in our meta-analysis of the *GSTP1* gene, and we were unable to explore the roles of other variants within the *GSTP1* gene, such as the Ala114Val polymorphism. The possible distinct effects of different haplotypes merits further evidence. In addition, the joint effects of *GSTM1, GSTT1, GSTP1*, and other genes, such as cytochrome P450 family 1 subfamily A member 1 (*CYP1A1*), is worthy of analysis upon publication of sufficient data.

No high degree of heterogeneity in the comparisons of the *GSTM1, GSTT1, and GSTP1* polymorphisms was detected in our study. For the meta-analysis of the *GSTP1* Ile105Val polymorphism, we included five case–control studies from four articles [12,15,16,19]. However, we observed obvious alteration of the pooled OR and 95% CI values when one study [16] was excluded during our sensitivity analysis. Therefore, we removed the present study and performed the meta-analysis again. We recognize that the data included in our meta-analysis were very limited.

Although we obtained a positive result for the role of the *GSTT1* present/null polymorphism in the risk of NP, the limitation of small sample size weakened the statistical power of our analysis to some extent. In addition, hospital-based controls were included in some studies. The genotype distribution of the *GSTP1* Ile105Val polymorphism in the control group of one article [12] did not agree with Hardy–Weinberg equilibrium. It would be of great value to thoroughly evaluate the influences of additional variables, such as clinical type, gender, age, environmental exposure or lifestyle, on the roles of the above variants in the risk of developing polyposis.

In summary, we pooled published relevant data and concluded that the *GSTT1* null genotype may serve as a protective factor against NP, whereas the Ile/Val genotype of the *GSTP1* Ile105Val polymorphism is more likely to be associated with an increased risk of NP. More genomic tests in the future are warranted to further determine the roles of glutathione S-transferase gene polymorphisms in the genetic susceptibility to different types of polyposis.

## Abbreviations

BICD2: BICD cargo adaptor 2
CI: confidence interval
CNKI: China National Knowledge Infrastructure
CP: colorectal polyposis
CYP1A1: cytochrome P450 family 1 subfamily A member 1
*GSTM1*: glutathione S-transferase M1
*GSTP1*: glutathione S-transferase pi
*GSTT1*: glutathione S-transferase T1
HLA-DRA: major histocompatibility complex, class II, DR α
HLCS: holocarboxylase synthetase
HWE: Hardy–Weinberg Equilibrium
JOAG: juvenile open-angle glaucoma
MeSH: medical subject heading
NP: nasal polyposis
OR: odds ratio
*P_A_*: *P*-value of the association test
*P_B_*: *P-*value of Begg’s test
*P_E_*: *P*-value of Egger’s test
*P_H_*: *P*-value of Cochran’s *Q* statistic test
PRISMA: Preferred Reporting Items for Systematic Reviews and Meta-Analyses
SLC5A1: solute carrier family 5 member 1
VSIR: V-set immunoregulatory receptor
WOS: Web of Science
